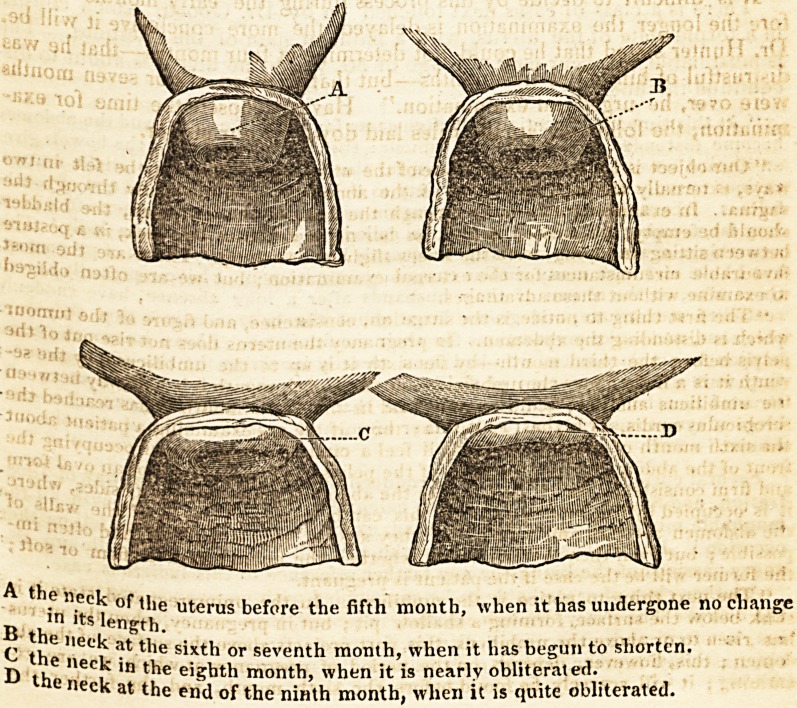# On the Mode of Distinguishing Pregnancy from the Diseases Which Resemble It

**Published:** 1830-01-01

**Authors:** 


### ON THE MODE OF DISTINGUISHING PREGNANCY FROM THE DISEASES WHICH RESEMBLE IT. By *Robert Gooch*, M. D.*

IN the recent and very talented work of Dr. Gooch, somewhat more than 50 pages, or nearly an eighth part of the whole book is occupied with the above-mentioned subject. It cannot be said therefore that we are overrating its importance by giving it a separate article in this Journal. The symptoms of pregnancy are considered by the world at large so unequivocal, that none but very ignorant people can mistake them—and yet they are often mistaken, not only by women and their nurses, but by medical men of ample experience.

"For want of clear notions of the subject, and a very attainable degree of tact, practitioners are frequently incurring disgrace, patients are subjected to active courses of medicine for the reduction of tumours, for which the natural remedy is parturition; and in some instances, pregnant women have been supposed to be dropsical, and actually tapped, to say nothing of other blunders." 199.

* An Account of Some of the Principal Diseases peculiar to Women. 1829.

Our able author thinks he shall do an essential service to a numerous class of practitioners, by giving a full and connected account of the symptoms of pregnancy—the degree in which they may be relied on—the mode of discriminating pregnancy from cases resembling it—and the various forms of disease to which it requires to be applied. The order or succession of pregnancy symptoms must be carefully attended to, as well as the symptoms themselves.

The first symptom is the interruption of the menses. When the period passes, morning sickness usually occurs, and generally continues during the first half of pregnancy, subsiding about the time of quickening. When three months have elapsed without menstruation, the abdomen begins to enlarge—the patient rather *feeling* distended than *shewing* any visible increase of size. After the fourth month, however, some degree of protuberance can be perceived. By the fifth month, no one can fail to perceive it, when the female is standing—and, from this time, it gradually increases till it attains the full size. Thus the visible enlargement does not last more than five months. If it has lasted nine months, that alone is a reason for doubting the existence of pregnancy.

The next symptoms in succession are enlargement and shooting pains in the breast—darkness of the areola—and swelling of the follicles around the nipples. The discoloration is very distinct in women who have dark hair and eyes—in those of a different complexion it is very equivocal.

The movements of the child. This is the most conclusive symptom, when it actually exists. These movements begin to be felt when four months have passed without menstruation. The sensation is, at first, very slight—like a pulse or fluttering in the abdomen, lasting only a few seconds. It may be felt once a day, and then cease for several—but gradually this sensation becomes stronger and more frequent, till at length if a hand happens to be laid on the abdomen at the moment when the child moves, it can be felt externally. Towards the end of pregnancy the movements are very strong, and the heavings of the abdomen may be seen through the dress.

If these symptoms always accompanied pregnancy, and never accompanied any other state, then we never could be in doubt; but, unfortunately, they may be absent in the impregnated, and present in the unimpregnated female.

In respect to *menstruation*, there can be no doubt that many women have a periodical discharge during pregnancy, so nearly resembling the ordinary secretion as not to be distinguished from it. The true nature of this discharge is not the object of inquiry here—it is sufficient to know that it exists.

*Sickness* varies so much in degree, kind, and duration, as to be quite fallacious. In thin women the *enlargement of the breasts* is often very slight—while in fat women the breast forms so small a proportion of the bosom that any enlargement of it is scarcely perceptible. In respect to the *areola*, it may be observed that, in very fair women, its discoloration is often so slight as to be with difficulty distinguished, while in brunettes, who have already borne children, the areola remains permanently dark.

"The enlargement of the abdomen from the third month to delivery, is in all cases present and progressive whilst the fœtus is alive; but it may die, and yet be retained till the ninth month, in which case the enlargement will not be progressive. The same may be said of the movement of the fœtus; it will not move if not alive, and there are cases, though rare, in which it has not moved during the whole of pregnancy, although it has been born alive and vigorous; of this I have known one instance, and read of others. Thus a woman may be pregnant though she seems to herself to continue to menstruate, has no sickness, or enlargement about the breasts, or darkness of the areola, or progressive enlargement of the abdomen, or perceptible movement of the fœtus. Such a complete assemblage of omissions however is not likely to meet in the same case." 204.

The fact is, that a woman may have all the symptoms of pregnancy, and yet not be pregnant. Menstruation may stop from other causes—sickness may arise from other causes—the bosom may enlarge because the female is growing plump—the abdomen may enlarge from flatulence, dropsy, or other diseases.

"As to the movements of the child, it is very important to distinguish between these movements as felt internally by the patient, and as felt externally by a hand applied to the surface of the abdomen; the latter, if really felt, is an infallible sign of pregnancy, but the former are often felt when there is no child. Thus a woman may cease to menstruate, have sickness, enlargement of the bosom, and darkness of the areola, a progressive enlargement of the abdomen, and sensations which resemble those produced by the movements of the child, without being pregnant." 206.

If the ordinary symptoms of pregnancy then be so little infallible, what is to be the result in practice? How are we to act in doubtful cases? We must wait till the doubtful state is sufficiently advanced to enable us to ascertain whether enlargement of the abdomen depends on enlargement of the uterus—and if so, whether the uterus contains a fœtus. These are the objects of the "examination by touch," and the several other indications are important only as they elucidate these two questions.

It is difficult to decide by this process during the early months—therefore the longer the examination is delayed, the more conclusive it will be. Dr. Hunter stated that he could not determine at four months—that he was distrustful of himself at five months—but that, "when six or seven months were over, he urged an examination." Having chosen the time for examination, the following are the rules laid down by our author.

"Our object is to ascertain the state of the uterus, and this may be felt in two ways, externally through the walls of the abdomen, and internally through the vagina. In examining externally through the walls of the abdomen, the bladder should be empty, the patient in bed, *in* her night dress, *on* her back, *in* a posture between sitting and lying, with the knees slightly drawn up. These are the most favourable circumstances for the external examination; but we are often obliged to examine without these advantages.

"The first thing to notice is the situation, consistence, and figure of the tumour which is distending the abdomen. In pregnancy the uterus does not rise out of the pelvis before the third month—by the sixth it is up to the umbilicus—by the seventh it is a little above the umbilicus—by the eighth month it is half-way between the umbilicus and scrobiculus cordis—and in the ninth month it has reached the scrobiculus cordis, its highest elevation; thus, if we are examining a patient about the sixth month of pregnancy we shall feel a circumscribed tumour occupying the front of the abdomen, from the brim of the pelvis to the umbilicus, of an oval form and firm consistency, much firmer than the abdomen above and on its sides, where it is occupied by the intestines. All this can be made out clearly if the walls of the abdomen are thin and relaxed; if they are fat, this is difficult, and often impossible; but even then we can notice whether the enlargement is firm or soft; the former will be the case if the patient is pregnant.

"The next thing to notice is the umbilicus. In the unimpregnated state it is sunk below the surface, forming a shallow pit; but in pregnancy, when the uterus has risen to or above the umbilicus, this part projects above the surface of the abdomen; this, however, depends on the period of pregnancy at which we are examining; it will scarcely be found before the sixth month, and the further the pregnancy is advanced the more distinct will it be. The firmness of the abdomen and the projection of the umbilicus depend on one and the same cause, that is, the firmness of the tumour which is distending the abdomen; but any other tumour equally firm may occasion both these symptoms; their presence alone proves little, but if the state which we are investigating is advanced as far as the seventh or eighth month, their absence proves a great deal, for if the umbilicus is depressed, and the abdomen, though enlarged, is soft and yielding, these alone prove that the patient is not pregnant. Let not the practitioner, however, give an opinion till he has collected all the proofs." 210.

The movement of the child is the next thing to be attended to. If the hand be laid on the naked abdomen, the fœtus will sometimes be felt to stir—and if distinctly so, the symptom is the most conclusive of all.

"Having examined the uterus through the walls of the abdomen, we proceed next to examine it through the vagina; for this the patient should be turned on her side; and here again there are three things to observe, the state of its neck, the state of its body, and the movement, or rather the mobility of the fœtus. 1st, in the unimpregnated state the neck of the uterus projects into the vagina about two-thirds of an inch, like a thick, firm, fleshy nipple. At the termination of pregnancy, a few days before labour, this neck is completely obliterated, the portion of uterus, which lies over the top of the vagina, no longer projecting into its cavity but forming a flat roof. This obliteration begins about the fifth month, the neck becoming gradually softer, broader, and shorter; by the seventh month it is much altered, and not at all like the neck in the unimpregnated state, being very soft, broad, and short. It is now calculated to have lost three-fourths of its length; but it is not quite obliterated till the last week of pregnancy, so that if a false alarm about labour, two or three weeks before delivery, gives the practitioner an opportunity of examining the uterus, he will find a soft short nipple still remaining." 213.

A the neck of the uterus before the fifth month, when it has undergone no change in its length.
B the neck at the sixth or seventh month, when it has begun to shorten.
C the neck in the eighth month, when it is nearly obliterated.
D the neck at the end of the ninth month, when it is quite obliterated.

The next thing is to ascertain whether the body of the uterus has been enlarged. This may be done by pressing up the finger between the neck of the uterus and the pubes, where, in the unimpregnated state, nothing will be felt but what is soft and yielding; but where pregnancy has advanced several months, this space is occupied by a large firm tumour. A practised hand finds no difficulty in detecting this difference—not so the inexperienced.

"There is a combination of the external and internal examinations, which in thin persons gives a very accurate knowledge of the nature of the tumour. For this purpose the finger of the right hand is to be applied against the tumour which is felt in the vagina, and the left hand is to be applied on the outside of the abdomen, to the upper part of the circumscribed swelling. Now by alternately pressing the tumour up, by means of the finger in the vagina, and down, by means of the hand on the abdomen, the practitioner becomes certain that the tumour which is felt through the walls of the abdomen, is the same as that which is felt through the vagina; the most satisfactory proof that it is an enlarged uterus. This method is applicable as early as the fourth or fifth month." 215.

As the fœtus floats in a bath of liquor amnii, its head rests over the top of the vagina, when the mother is in the erect position. If the practitioner applies his finger to the uterus just in front of the neck, and gives it a push, the fœtus will float for an instant, and the next instant fall with perceptible weight on the point of the finger. This sensation, if once felt, can never be forgotten. It is scarcely inferior to the muscular movements of the child —and has this advantage, that it can be felt whether the fœtus be dead or alive. The best period for this species of proof is between the fifth and seventh month.

There is a class of cases next touched on by Dr. Gooch, which consists in a torpid state of the uterus, with a flatulent state of the intestines. This is most liable to occur near the age of 50, when the uterus is about to discontinue its functions.

"At this time menstruation will often cease for several months, and the abdomen become distended with a flatulent tumour: the air moving about the bowels gives an inward sensation which is mistaken for the child; there is often slight nausea, various nervous feelings, and an anxiety to believe in pregnancy as a test of youthfulness. About this age, also, the omentum and parietes of the abdomen often grow very fat, forming what Dr. Baillie once called 'a double chin in the belly.' This assemblage of symptoms at this age frequently leads to the supposition of pregnancy, but I have met with many similar cases in young women. I have repeatedly known those who, on the return of their husbands after a long absence, have suddenly ceased to menstruate, and grown large about the belly, conclude that they were pregnant, and make preparations for their confinement. I have known the same happen to single women, who had been secretly incurring the risk of pregnancy; they were generally women of sickly constitutions, who were very subject to obstructed menstruation; and it is probable that in these cases the puzzling assemblage of symptoms was the result rather of mental agitation than of sexual intercourse." 226.

Several curious cases are here introduced by our author, in the way of illustration, and among others that of the celebrated Joanna Southcott, with all the laughable mistakes into which some sad, grave, and learned doctors fell on that occasion! But we shall not rake up the ashes of the dead, nor kindle up the angry passions of the living, by any farther reference to this ludicrous case.

Another class of cases liable to be confounded with pregnancy, are tumours of the ovary. The ovary, though smaller, in health, than the unimpregnated uterus, is often more enlarged in disease, forming, like the impregnated womb, a circumscribed tumour, which rises out of the pelvis to various heights in the abdomen. These tumours are sometimes mistaken for pregnancy—but hardly by a competent judge. The duration of the tumour, which is generally much beyond that of pregnancy, is alone a sufficient guide—though *one* circumstance should never be trusted to. The examination per vaginam settles the question. When a large ovary is mistaken for pregnancy, the error is comparatively harmless—but when pregnancy is mistaken for ovarian dropsy, and paracentesis is performed, the matter is serious. Dr. Gooch has heard of several such mistakes. But a woman with ovarian disease may become impregnated. Our author has seen several instances of this kind. The examination per vaginam is still the only safe mode of ascertaining the real state of the case.

But sometimes the cause which distends the abdomen is inside of the uterus, and not a fœtus—viz. air, water, hydatids. Dr. Gooch has never seen an instance of tympanitis uteri, neither has he ever met with one of dropsy of the uterus; but there are many on record. The one related by Dr. Thomson, in the Medico-Chirurgical Transactions, is a recent example. Of hydatids in the uterus Dr. Gooch has, of course, met with many cases. The patients had the ordinary symptoms of pregnancy, only with some peculiarity which led them to doubt it—such as the absence of fœtal movements, the enlargement of the abdomen being disproportionate to the period of pregnancy, or, after advancing rapidly becoming suddenly stationary. In other cases, the patient, after supposing herself pregnant, had a discharge, sometimes of blood and sometimes of water, leading her to suppose that she was miscarrying. A short case may here be introduced.

"I was sent for to ———, a few miles from London, to see a lady, who having ceased to menstruate for one month, and becoming very sick, concluded that she was pregnant; the next month she had a slow hæmorrhage from the uterus, which had continued incessantly a month when I saw her; she kept nothing on her stomach. On examining the uterus through the vagina its body felt considerably enlarged, and there was a round circumscribed tumour in the front of the abdomen, reaching from the brim of the pelvis nearly to the umbilicus. I saw her several times at intervals of a fortnight, during which the hæmorrhage and the vomiting continued unrelieved; the peculiarity about the case was the bulk of the uterus, which was greater than it ought to be at this period of pregnancy; it felt also less firm than the pregnant uterus, more like a thick bladder full of fluid. Eleven weeks from the omission of menstruation she was seized with profuse hæmorrhage; towards evening there came on strong expelling pains, during which she discharged a vast quantity of something which puzzled her attendants. The next morning I found her quite well, her pain, hæmorrhage, and vomiting having ceased. I was then taken into her dressing-room and shown a large wash-hand basin full of what looked like myriads of little white currants floating in red currant juice; they were hydatids floating in bloody water." 244.

In the progress of these cases Dr. G. believes it impossible to come nearer to the truth than this:—that the abdomen owes its enlargement to a distended uterus, but what this organ contains is uncertain. The following case was, at first, supposed to be pregnancy—afterwards hydatids—and turned out to be neither one nor the other.

"A lady, the mother of a large family, having ceased to menstruate for several months, and growing large in the abdomen concluded that she was with child—at length there came on a profuse and perpetual discharge of water, sometimes mixed with blood, by which her strength was so alarmingly reduced, that first one and then another practitioner was consulted about her, and I met a consultation of four. Through the walls of the abdomen, the uterus could be felt, about as high as the umbilicus, and in the vagina the neck of the uterus was found obliterated, and its body enlarged. As every attempt to restrain the discharge and support her strength had been unavailing, and she daily became more exhausted, a silver tube was introduced through the orifice of the uterus into its cavity, that if it was distended by an ovum, the liquor amnii might be drawn off. The tube readily passed in, but on withdrawing the wire no liquor amnii came away. A few hours afterwards she was seized with violent expulsive pains, under which she sunk rapidly and died. I was not present at the examination of the body; but the following statement was sent me by the gentleman who opened it. The uterus was as large as at the sixth month of pregnancy, and its cavity big enough to hold two fists; it contained neither fœtus nor hydatids, but a mass about the size of a goose's egg of stringy matter, like very soft placenta and unattached to the inner surface of the uterus; this surface was red and irregular, like a granulating sore; its walls were thickened as in pregnancy, of a dark red hue, and a flaccid texture." 247.

Dr. Baillie has alluded to something of this kind, in the following terms.

"It sometimes happens," says Dr. Baillie, "though not very often, that the uterus enlarges in size, and becomes much harder than in its natural state. This change corresponds very much to that of scirrhus in other parts of the body, and commonly extends over the whole of the uterus. I have seen it in one case as large as the gravid uterus at the sixth month; ulceration, I believe, is commonly wanting." 247.

The last case in this section of our author's work we shall endeavour to abridge, though Dr. Gooch says he cannot abridge it. Let us try.

A farmer's wife, aged 45, married 20 years without children, had been jolted in a cart, three years previous to her application, after which profuse menstruation came on, and continued more or less ever since, inducing debility and emaciation. Eighteen months ago, the abdomen began to enlarge—the enlargement increasing ever since, but latterly more rapidly. On examination of the sides, the space between the ribs and ilium felt soft and flatulent, the front o the abdomen being occupied by a circumscribed tumour of stony hardness. The umbilicus projected—the cervix uteri was broad, and short as in the seventh month. The space between the cervix and uterus was occupied by a very hard tumour. Here were all the symptoms of pregnancy, yet the woman was not pregnant. The result was not ascertained.

Our author has thus fulfilled the object of his paper, and observes that, if the young practitioner will make himself master of the detail, and acquire a little familiarity with the feel of the uterus in the pregnant and unpregnant states, the said detail will generally guide him right. Some have said that this art of distinguishing between the two states is a blind tact, gained only by practice, and incommunicable by instruction. To this our author does not agree.

"The period of my life when I improved most rapidly in the art of deciding by examination cases of doubtful pregnancy, was that in which I gained clear and orderly notions of the objects of examination. The faculty of observation requires rather to be guided, than to be sharpened; the finger soon gains the power of feeling, when the mind has acquired the knowledge of what to feel for." 249.

We too have fulfilled our object, and, in five or six pages of our Journal, have condensed the pith or marrow of 50 pages of goodly letter-press. We have taken a leaf out of Dr. Gooch's book—or at least a hint. It is this:—"provided you get the *points* of the work, the more briefly you do it the better:—if you are skilful at this, you will find that a page will hold a pamphlet—and 20 pages will often hold a bulky volume."—*Preface.* We are inclined to flatter ourselves that experience has conferred on us some *skill* in the art of condensing; but, in justice to Dr. Gooch, we aver that no degree of skill, not even that of Richter, whom he recommends as a model, could enable a writer to compress the matter of Dr. G's book into such spaces as he talks of in the above passage.

## Figures and Tables

**Figure f1:**